# Value-based outsourcing is associated with better environmental and economic sustainability metrics while maintaining patient safety: a comparative analysis of electricity use, water use, and prescription costs in public hospitals of Madrid (Spain)

**DOI:** 10.3389/frhs.2026.1762450

**Published:** 2026-04-15

**Authors:** Marta del Olmo Rodríguez, Bernadette Pfang, Ángel Blanco Rubio, David Romera, Ruben Horcajo, Javier Becares, Javier Arcos, Juan Antonio Álvaro de la Parra, Jorge Short Apellaniz

**Affiliations:** 1 Quirónsalud 4H Network, Madrid, Spain; 2 University Hospital Fundación Jiménez Díaz, Madrid, Spain; 3 Instituto de Investigacion Sanitaria de la Fundacion Jimenez Diaz, Madrid, Spain; 4 Quirónsalud Healthcare Network, Madrid, Spain; 5 Hospital General de Villalba, Collado Villalba, Spain

**Keywords:** economic sustainability, healthcare and environment, healthcare policy and management, sustainable healthcare, value-based healthcare (VBHC)

## Abstract

**Background:**

Healthcare systems face growing pressure to maintain high-quality care while improving environmental and economic sustainability. Hospitals are resource-intensive institutions, contributing substantially to energy and water consumption, pharmaceutical expenditure, and healthcare-related greenhouse gas emissions. Value-based healthcare models, including value-based outsourcing of publicly funded hospitals to private networks, have shown promise in improving quality and efficiency. However, their impact on sustainability outcomes remains underexplored. This study compared environmental and economic sustainability indicators between value-based outsourced and publicly managed hospitals.

**Methods:**

We conducted a cross-sectional comparative study using five years of administrative data (2019–2023) from 26 public hospitals in the Madrid Regional Healthcare Service, Spain. Four hospitals were managed by a value-based private network, and 22 were publicly managed. Outcomes included electricity consumption (kWh/m^2^ per hospital stay), water consumption (m^3^ per hospital stay), and mean unit cost of specialist-prescribed medications. Analyses were stratified by hospital complexity (low, medium, high) and adjusted for case-mix complexity and year of construction. Patient safety indicators (medical and surgical complications and hospital-acquired infections) were also compared. Statistical analyses used chi-squared, Student's *t*-test, or Mann–Whitney *U* tests, with *p* < 0.05 considered significant.

**Results:**

Outsourced hospitals treated patients with significantly higher overall case-mix complexity than publicly managed hospitals. Overall electricity consumption was lower in outsourced hospitals (0.009 vs. 0.011 kWh/m^2^ per hospital stay; *p* = 0.039), leading to estimated cost savings of 1,648,305 € and an approximate reduction in greenhouse gas emissions of 8,672 CO2 metric tons during the study period, as was water consumption (0.65 vs. 0.81 m^3^ per hospital stay; *p* < 0.001). The mean unit cost per medication was also lower in outsourced hospitals (€21.45 vs. €22.92; *p* = 0.026), with significant differences primarily in medium-complexity hospitals. Outsourced hospitals demonstrated slightly but significantly lower rates of inpatient complications and hospital-acquired infections.

**Conclusions:**

Value-based outsourcing in Madrid's public hospitals was associated with improved environmental and economic sustainability, alongside favorable patient safety outcomes, despite higher case-mix complexity. These findings support incorporating sustainability indicators into performance-based healthcare models and suggest value-based outsourcing as a potential strategy to advance sustainable healthcare delivery.

## Strengths and limitations

- System-wide comparison including all public hospitals in Madrid.
- Reliable, standardized administrative data.
- Cross-sectional design limits causal inference.
- Environmental metrics restricted to electricity and water use.

## Introduction

Healthcare systems worldwide are under increasing pressure to deliver high-quality care while addressing climate and economic sustainability challenges (1). Hospitals are among the most resource-intensive public institutions, consuming large amounts of electricity and water and accounting for substantial pharmaceutical expenditures, as well as around one-third of healthcare-associated greenhouse gas emissions (2, 3). Rising demand, budgetary constraints, and climate change-related threats require innovative models of healthcare delivery that balance efficiency, equity, and sustainability (4).

Value-based healthcare is a model of care which seeks to provide the highest value for patients at the lowest possible cost (5). As well as in the United States, several value-based healthcare initiatives have been implemented in European contexts, with evidence pointing towards positive results regarding quality of care and cost reduction (6–11). In particular, value-based outsourcing, in which publicly owned and funded hospitals are outsourced to a value-based private healthcare network, is a promising strategy with initial evidence pointing to positive outcomes regarding quality of care, efficiency and patient satisfaction (12–14). However, the impact of value-based outsourcing on sustainability metrics has not been thoroughly investigated.

Sustainability in healthcare is a complex concept which includes both environmental sustainability—such as responsible consumption of electricity and water—and economic sustainability. In Spain, pharmaceutical expenditure accounts for around 20% of total healthcare spending (15), making the optimization of prescriptions a major concern (16). Similarly, hospitals are major consumers of electricity and water, with environmental implications that extend beyond the health system (17–20).

To our knowledge, no previous study has directly compared the performance of value-based outsourced hospitals and publicly managed hospitals in terms of sustainability outcomes. The objective of this study is to assess whether value-based outsourcing is associated with better performance regarding indicators of environmental and economic sustainability, including electricity consumption, water consumption, and specialist prescription costs.

## Methods

### Study design and setting

This was a cross-sectional comparative study including six years of data (2019–2024) from 26 public hospitals in Madrid, Spain. All hospitals are part of the Regional Madrid Healthcare Service and—regardless of management status—provide universal care at zero out-of-pocket cost to patients in their catchment areas, as well as patients choosing to transfer from other catchment areas under the 2011 Free Choice of Care Act. Of these 26 public hospitals, four are managed by the value-based Quirónsalud healthcare network (the study group), one is managed by a traditional for-profit healthcare network while the remaining 21 are managed directly by the regional health authority.

### Participants

We included all public hospitals from the Madrid healthcare system, excluding long-term facilities, exclusively pediatric hospitals and psychiatric hospitals. Our study group included the four hospitals outsourced to a value-based private network, comprising one low-complexity hospital (Infanta Elena University Hospital), two medium-complexity hospitals (General Villalba University Hospital and Rey Juan Carlos University Hospital), and one high-complexity hospital (Fundación Jiménez Díaz University Hospital). The other 22 hospitals were the control group.

### Patients and the public

Patients were not actively involved in the study design. However, the public hospitals of Madrid (Spain) provide universal coverage to inhabitants of the Madrid province, and environmental and economic sustainability along with safety metrics are of interest to public health.

### Data sources

Data were extracted from annual hospital reports of the Regional Healthcare Service of Madrid (RHSM) from 2019 to 2024. These annual reports gather and analyze data from the different hospitals belonging to the RHSM through a series of centralized databases. Staff from the Regional Outcomes Observatory of the RHSM have access to raw data and independently analyze data to compare hospital performance across a range of indicators (21). These results are then made available to the public in yearly reports published via the Regional Outcomes Observatory's webpage. Our study featured a subset of publicly available data published in annual reports.

### Outcomes and measurements

The primary objectives of the study were to compare use of electricity and water between hospitals from the study and control groups, as well as the mean unit cost of prescribed medications. We extracted annual electricity consumption from RHSM annual reports, in which electricity consumption was reported as total annual electricity consumption (kWh) per square meter and hospital stay in order to normalize data according to hospital size and patient load; data were retrieved by the Regional Outcomes Observatory staff from each hospital's yearly electricity bills and the centralized database for mandatory discharge data. Water consumption was reported in RHSM annual reports as m^3^ per hospital stay, and was retrieved from yearly utility bills, while the total number of hospital stays per year for each center was obtained using the centralized database for mandatory hospital discharge data, as reported in RHSM annual reports.

Regarding economic sustainability, the cost of medications represents a significant proportion of the Madrid Healthcare System's budget and, as such, is closely monitored. Patients must receive the necessary medicines to manage their conditions; however, for the same therapeutic objective, multiple alternatives may exist with comparable efficacy and safety but different prices. The mean unit cost of prescribed medications as calculated by RHSM annual reports reflects the average cost of medication prescribed by specialist physicians and dispensed to patients at their local pharmacies, where a lower unit cost indicates a more efficient use of healthcare resources.

To account for potential bias due to differences in construction and case-mix complexity between hospitals, we also included the year of construction of each hospital and performed an analysis of case-mix complexity between hospitals in the study and control groups. Mean case-mix complexity for hospital discharges was extracted from the RHSM annual reports, where it serves as a management indicator reflecting the average clinical complexity of patients treated within a facility, quantified by resource consumption. Each inpatient episode is categorized into a Diagnosis-Related Group (DRG) based on variables such as primary diagnosis, secondary diagnoses, surgical procedures, age, sex, and discharge status. DRGs constitute a classification system for acute hospitalization episodes, aggregating cases that are clinically homogeneous and characterized by similar expected resource utilization. Each DRG is assigned a relative weight representing the expected cost of managing such patients in relation to the average cost of all acute hospitalizations; these weights are traditionally calculated annually in the United States. A higher case-mix complexity signifies a greater level of complexity among the patient population served.

Although previous studies have observed superior healthcare outcomes for the study hospitals, we also included an analysis of two patient safety indicators (medical and surgical complications during hospital stay, and hospital-acquired infections) to control for potential differences in these variables associated with differences in sustainability indicators between groups. The RHSM calculated annual rates for inpatient surgical and medical complications by dividing the number of patients discharged with at least one coded complication by the total number of annual discharges, expressed as a percentage. To assess the prevalence of hospital-acquired infections (HAIs), each facility conducted point-prevalence surveys based on the standardized European Point Prevalence Survey (EPPS) methodology (22). These results were extracted from the RHSM annual reports for 2021–2024 (no data were available for 2020 due to the COVID-19 pandemic). To facilitate comparison between study groups, the point-prevalence infection rates were extrapolated by multiplying them by the total number of inpatient stays recorded during the study period.

To estimate the number of KWh saved during the study period, we used this equation:

$$\begin{array}{rl} \mathrm{Total}\;\mathrm{kWh}\;\mathrm{saved} & =\left({\frac{\frac{\mathrm{kWh}}{m^{2}}}{\mathrm{stay}}}_{\mathrm{study}\;\mathrm{group}}\;-{\frac{\frac{\mathrm{kWh}}{m^{2}}}{\mathrm{stay}}}_{\mathrm{control}\;\mathrm{group}}\right)\\ & x\;(\mathrm{total}\;\mathrm{surface}\;\mathrm{are}a_{\mathrm{study}\;\mathrm{group}})\\ & x\;(\mathrm{total}\;\mathrm{patient}\;\mathrm{stay}s_{\mathrm{study}\;\mathrm{group}}) \end{array}$$

We used the EPA Greenhouse Gas Equivalencies Calculator to convert KWh into CO2 metric tons (23), and the average price per KWh for 2024 (0.1277€/KWh) (24).

### Statistical analysis

Categorical variables were reported as number ± frequency, and continuous variables as average ± standard deviation. Prevalence of medical and surgical inpatient complications and healthcare-acquired infection between groups were compared using a Chi-squared test, after transforming the variables by multiplying by number of inpatients per year. Continuous variables were compared using a Student's *T*-test or a Mann–Whitney *U* test, according to data distribution. Analyses were performed for each stratum (low complexity, medium complexity, and high complexity) and overall. A *p*-value of less than 0.05 was deemed statistically significant. Analyses were performed using Python version 3.1.

### Ethics

This study used aggregated, anonymized administrative data and did not require individual patient consent. The study received a formal waiver for review by the Fundación Jiménez Díaz Ethics Committee.

## Results

The analysis included 26 hospitals, of which four were outsourced and 22 publicly managed. Data regarding year of inauguration, number of beds, and sociodemographic characteristics of the hospital catchment areas are presented in Table 1. Overall, no significant differences in average annual hospital admissions per year were observed between groups (Table 2). On average, the study group hospitals attended a patient population with significantly higher case-mix complexity during the study period than hospitals from the control group (0.94 ± 0.11 vs. 0.85 ± 0.13, CI 0.03–0.14, *P* = 0.004).

**Table 1:** Characteristics of hospitals included in the analysis.

Hospital	Municipality	Beds	Approximate surface area (m^2^)	Inauguration
High-Complexity Hospitals				
Control 16^a^	Madrid	861	166,357	1951
Control 17^a^	Madrid	1,140	172,000	1968
Control 18 (25)	Madrid	1,161	148,262	1973
Control 19^a^	Madrid	524	68,621	1955
Control 20^a^	Madrid	966	190,842	1964
Control 21 (26)	Majadahonda	613	165,000	2008
Control 22^a^	Madrid	886	150,000	1977
Study hospital 4 (27)	Madrid	651	76,500	1955
Medium-Complexity Hospitals				
Control 6	Madrid	473	Information not found	1896
Control 7	Fuenlabrada	413	Information not found	2004
Control 8	Getafe	543	Information not found	1991
Control 9 (28)	Móstoles	328	44,142	1983
Control 10 (29)	Torrejón de Ardoz	250	52,959	2011
Control 11 (30)	Alcorcón	401	112,708	1997
Control 12 (31)	Madrid	269	85,066	2008
Control 13^a^	San Sebastián de los Reyes	276	84,290	2008
Control 14 (32)	Alcalá de Henares	507	81,930	1987
Control 15^a^	Leganés	386	58,000	1987
Study hospital 2 (33)	Collado Villalba	217	69,066	2014
Study hospital 3 (34)	Móstoles	328	89,413	2012
Low-Complexity Hospitals				
Control 1^a^	San Lorenzo de El Escorial	92	24,774	1995
Control 2 (35)	Coslada	249	75,800	2008
Control 3 (36)	Arganda del Rey	132	45,000	2008
Control 4 (37)	Aranjuez	102	46,000	2008
Control 5 (38)	Parla	188	56,811	2008
Study hospital 1 (39)	Valdemoro	186	53,271	2007

a Information taken from annual hospital reports.

**Table 2:** Baseline characteristics of study and control hospitals.

Average hospital admissions/year ± SD		
Low complexity		
Study group	Control group	*P*-value
10,397 ± 1,112	7,066 ± 2,879	<0.001
Medium complexity		
Study group	Control group	*P*-value
15,648 ± 5,210	15,173 ± 2,732	0.773
High complexity		
Study group	Control group	*P*-value
28,159 ± 1,992	33,736 ± 10,803	0.006
Overall		
Study group	Control group	*P*-value
17,463 ± 7,589	19,268 ± 12,306	0.351
Average case-mix complexity		
Low complexity		
Study group	Control group	*P*-value
0.99 ± 0.09	0.83 ± 0.09	0.016
Medium complexity		
Study group	Control group	*P*-value
0.99 ± 0.08	0.80 ± 0.08	0.006
High complexity		
Study group	Control group	*P*-value
1.05 ± 0.11	0.87 ± 0.10	0.012
Overall		
Study group	Control group	*P*-value
1.03 ± 0.12	0.93 ± 0.15	0.004
Year of inauguration		
Low complexity		
Study group	Control group (median, range)	
2007	2008 (1990–2008)	
Medium complexity		
Study group	Control group (median, range)	
2012, 2014	2000 (1978–2014)	
High complexity		
Study group	Control group (median, range)	
1955	1966 (1955–2008)	

Regarding electricity consumption, overall, significantly lower average consumption was observed for the outsourced hospitals (0.0088 kWh/m^2^/hospital stay vs. 0.011 kWh/m^2^/hospital stay, *P* = 0.018) (Table 3). We estimated savings of 12,907,632 kWh during the study period for the study hospitals compared to control hospitals, translating as cost-savings of 1,648,305€ and a reduction of 8,672 metric tons of CO2.

**Table 3:** Environmental and economic sustainability indicators for study and control groups, 2019–2024.

Average yearly electricity consumption (kWh/m^2^/hospital stay)		
Low complexity		
Study group	Control group	*P*-value
0.0116 ± 0.0014	0.0238 ± 0.0090	<0.001
Medium complexity		
Study group	Control group	*P*-value
0.0081 ± 0.0022	0.0086 ± 0.0028	0.560
High complexity		
Study group	Control group	*P*-value
0.0073 ± 0.0006	0.0052 ± 0.0020	<0.001
Overall		
Study group	Control group	*P*-value
0.0088 ± 0.0024	0.0110 ± 0.0086	0.018
Average yearly water consumption (m^3^/hospital stay)		
Low complexity		
Study group	Control group	*P*-value
0.73 ± 0.05	0.81 ± 0.11	0.030
Medium complexity		
Study group	Control group	*P*-value
0.69 ± 0.13	0.88 ± 0.36	0.003
High complexity		
Study group	Control group	*P*-value
0.45 ± 0.02	0.72 ± 0.12	<0.001
Overall		
Study group	Control group	*P*-value
0.64 ± 0.15	0.81 ± 0.26	<0.001
Average unit price per specialist-prescribed medication (€/unit)		
Low complexity		
Study group	Control group	*P*-value
19.31 ± 0.82	19.78 ± 1.14	0.348
Medium complexity		
Study group	Control group	*P*-value
19.79 ± 0.80	21.49 ± 1.51	<0.001
High complexity		
Study group	Control group	*P*-value
23.81 ± 0.57	24.21 ± 1.67	0.346
Overall		
Study group	Control group	*P*-value
20.68 ± 1.97	21.96 ± 2.23	0.015

Annual water consumption per patient was lower in outsourced hospitals compared with publicly managed hospitals (0.64 ± 0.15 m^3^/hospital stay vs. 0.81 ± 0.25 m^3^/hospital stay, CI −0.25 to −0.08, *P* = <0.001) when comparing both groups. When comparing each stratum, consumption was significantly lower for the medium and high complexity groups, but no significant differences were found for the low complexity group (Table 3).

The mean price per medication unit was lower in outsourced hospitals (20.68€ ± 1.97 €/unit) compared with publicly managed hospitals (21.96€ ± 2.23 €/unit, *p* = 0.015), although this difference was only significant for the medium complexity group upon stratum analysis (Table 3).

Finally, the two patient safety indicators included in our analysis demonstrated slightly lower rates of surgical and medical inpatient complications (3.11% vs. 3.89%, *P* < 0.001) and lower rates of hospital-acquired infection (4.19% vs. 7.06%, *P* < 0.001) for the four study group hospitals.

## Discussion

Our analysis of six years of data from the regional Madrid Healthcare Service (Madrid, Spain) demonstrates that hospitals outsourced to a value-based healthcare network outperformed publicly managed hospitals regarding sustainability metrics, including electricity consumption, water consumption, and cost per medication unit, while maintaining better patient safety metrics (hospital-acquired infection and medical and surgical inpatient complications). Notably, the study hospitals attended significantly higher numbers of patients per year with a significantly more complex case-mix than their publicly managed counterparts. Overall, our findings suggest that value-based outsourcing is not only associated with improved clinical performance, as previously demonstrated, but may also confer environmental and economic advantages.

Hospitals are among the largest public energy consumers due to their 24-h operations, reliance on high-powered clinical equipment, and the need for highly controlled environments involving heating, ventilation, air conditioning, lighting, and water systems (1, 40). This makes them complex, energy-intensive buildings with varying requirements across different areas, from high-demand surgical units to lower-demand administrative sectors (41). While many organizations have long incorporated sustainability into their business models, hospitals have traditionally been slow adapters regarding more efficient energy use in the context of environmental sustainability (2, 42, 43). Effective energy management—focusing on efficiency—can reduce consumption, emissions, and costs, while enhancing competitiveness. However, challenges remain, including the need to balance reduced energy consumption with maintaining care quality, high operating costs, patient unawareness of efficient practices, and the significant investments required for advanced energy-efficient technologies (1, 2, 43). Consequently, hospitals face unique difficulties in designing, operating, and managing energy systems, underscoring the urgency of implementing sustainable energy strategies in healthcare.

In our study, the hospitals in the study group performed better on average than publicly managed hospitals regarding electricity and water consumption. While statistically significant, the study group results regarding electricity demonstrate a slight difference which has a minimal impact on carbon emissions [approximately 10.1 metric tons per year (44)] and overall cost savings. However, water savings are indeed environmentally significant with an average of and while it is probable that hospital age and infrastructure are important factors contributing to differences observed in the subgroup analysis of electricity consumption, we hypothesize that value-based outsourcing also aligns management with performance and efficiency targets, thus incentivizing sustainability. For example, while the yearly budget assigned to publicly managed hospitals does not include costs associated with electricity, water, and other utility bills (which are covered by the Madrid Regional Healthcare Service under a separate allowance), the four study hospitals are reimbursed through a capitation model, with a fixed payment ceiling which must cover not only costs of services provided, but also other healthcare delivery-associated costs such as utilities, maintenance, and financing services. This acts as a strong incentive to implement sustainable practices across all areas of healthcare delivery, from surgical facilities to outpatient clinics. At the same time, a systematic review of hospital environmental sustainability concluded that preventive or demand management measures to avoid unnecessary procedures were more likely to improve hospital-associated climate impact than changes in how procedures were performed (18). In this regard, the study hospitals have implemented various initiatives to reduce unnecessary tests and hospital visits, thus reducing unnecessary consumption of resources and lowering environmental impact while improving patient safety and quality of life (45–50).

Possible drawbacks of re-organizing water and electricity consumption to improve environmental sustainability include potential safety concerns and reduced patient experience (2). While a previous Spanish-based study demonstrated lower water consumption per bed for privately managed hospitals outsourced to different healthcare networks (51), this study adds information on safety metrics for inpatients, suggesting that value-based outsourcing is associated with improved economic and environmental performance while maintaining high standards of patient safety.

Regarding economic sustainability, the unit price per medication prescribed by hospital specialists was significantly lower for the study group, plausibly indicating more cost-effective practices in outsourced hospitals. Our results are in line with a recent systematic review which demonstrated that continuous professional development for drug prescribing was associated with improved effectiveness and reduced healthcare costs (52). While our study did not include data on outpatient clinical outcomes, research shows that up to one-third of prescriptions are inappropriate (53, 54), potentially causing worse clinical outcomes as well as unnecessary healthcare expenditure (55–57).

Strengths of this study include its inclusion of all public hospitals in Madrid and the reliability of the source of data which comes from a public administration database, allowing the comparison of a wide range of healthcare indicators. Limitations include the cross-sectional design, which precludes causal inference, as well as potential confounding due to hospital age and infrastructure differences. Additionally, the analysis did not capture broader environmental indicators such as carbon footprint or waste generation.

Our findings suggest that sustainability should be explicitly considered in the evaluation of healthcare delivery models. Value-based outsourcing may represent a viable approach for health systems aiming to simultaneously improve efficiency, clinical outcomes, and environmental responsibility. Future studies should adopt prospective designs to assess the persistence of sustainability benefits over time and expand the scope of environmental metrics, including carbon emissions and waste reduction. Finally, similar studies in diverse healthcare would further elucidate the generalizability of these findings.

## Conclusions

Value-based outsourcing in Madrid's regional hospitals is associated with improved sustainability outcomes, including reduced electricity and water consumption and lower specialist prescribing costs. These results support the integration of sustainability indicators into performance-based healthcare models and highlight the potential of value-based outsourcing to contribute to environmentally and economically sustainable health systems while maintaining high standards of patient safety.

## Data Availability

The original contributions presented in the study are included in the article/Supplementary Material, further inquiries can be directed to the corresponding author.
